# Mesenteric Ischemia and Its Need for Timely Recognition and Management

**DOI:** 10.1155/2022/7370634

**Published:** 2022-09-28

**Authors:** Anish Kumar Shrestha, Anisha Shrestha, Bikal Ghimire, Prajwal Ram Ghimire, Simin Kunwar, Prajjwol Luitel, Niranjan Adhikari

**Affiliations:** Department of GI and General Surgery, Maharajgunj Medical Campus, Institute of Medicine, Tribhuvan University Teaching Hospital, Nepal 44600

## Abstract

Mesenteric ischemia is a fatal vascular emergency of the small intestine which, if not diagnosed and treated in time, has a very high mortality rate. Presenting with nonspecific symptoms such as abdominal pain, nausea, constipation, tachycardia, and gastrointestinal bleeds, it can masquerade as other causes of acute abdomen, particularly bowel obstruction. Ideal laboratory tests and markers are still lacking due to complexity in bowel's anatomy, physiology, blood supply, and drainage. We report 10 cases of mesenteric ischemia presented in our center with their demography, laboratory findings, approach to diagnosis, and treatment along with their outcomes at discharge. Out of the ten cases, six cases presented with signs and symptoms of acute bowel obstruction without passage of stool and one with passage of black stool. These seven patients underwent laparotomy, while the remaining three cases who either presented with milder symptoms or refused surgical interventions were managed conservatively. All patients were diagnosed with either acute or chronic mesenteric ischemia based on their operative and/or radiographic findings.

## 1. Introduction

Mesenteric ischemia is a potentially fatal vascular emergency with a mortality rate of about 60-90% [1–4] which results due to interruption or decrease in splanchnic-mesenteric blood flow causing transmural bowel wall necrosis ultimately leading to gangrene, peritonitis, sepsis, intra-abdominal free air, multiorgan failure, and death [5, 6]. Mesentery is supplied by the splanchnic circulation and receives approximately 25% of resting cardiac output [2, 5, 7]. Due to the complexity in its anatomy, physiology, blood supply, and drainage, ideal diagnostic lab tests and imaging techniques are still not sensitive for timely detection and intervention. It is because of this reason, despite knowing that time is everything in this disease, the overall survival has changed very little in the past 35 years [1].

The aim of our study is to describe the demography, presenting features, diagnostic approach, treatment, and outcomes of patients diagnosed with mesenteric ischemia.

## 2. Methodology

10 patients of both the sexes with postoperative and/or radiological diagnosis of mesenteric ischemia presenting in our center, in the Department of GI and General Surgery, within a year interval were included in this case series. This is a single-center case series. This series was conducted in a retrospective fashion reviewing patient's files. Demographic data like age and sex were analyzed. Any existing cardiac disease diagnosed either via electrocardiography (ECG) or echocardiography (ECHO) was taken into consideration as major risk factors for embolism. Basic laboratory parameters including total leukocyte count or white blood cell (WBC) count, pH, and lactate were analyzed in order to evaluate their roles in supporting the diagnosis of mesenteric ischemia as well as establishing the severity of the disease. Then, either a radiological or postoperative diagnosis was made and correlated with the treatment they received. Finally, the outcome was compared at the time of discharge of the patients.

All these data were analyzed using statistical tools like Microsoft Excel.

## 3. Cases

### 3.1. Case 1

**Figure 1:**
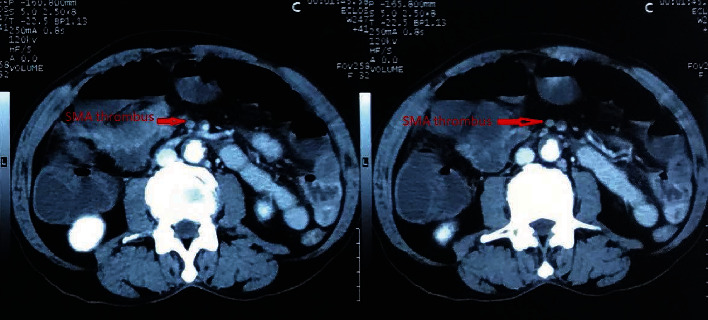
CT scan depicting SMA thrombosis.

A 76-year-old man was admitted to the hospital with a history of sudden-onset abdominal pain, initially periumbilical in location with generalization later, accompanied by vomiting and no passage of stool for 2 days. On examination, irregular pulse was noted. Abdominal examination revealed mild tenderness in the central abdomen and an absence of bowel sound. Laboratory investigations were normal; however, ECG revealed atrial fibrillation with a ventricular rate of 99/min, and arterial blood gas (ABG) report was normal except increased lactate level of 2. CECT of the abdomen suggested an occlusion of superior mesenteric artery (Figure 1). The patient was rushed immediately for surgery with the diagnosis of complete bowel obstruction with SMA thrombosis with atrial fibrillation (AF), and thereby, exploratory laparotomy with resection and anastomosis was performed. Intraoperative findings were gangrenous bowel 70 cm distal from duodeno-jejunal (DJ) flexure to hepatic flexure and mild ascites. Postoperatively, the patient showed signs of improvement at the beginning but later developed complications of anastomosis leakage which ultimately led to the demise of the patient.

### 3.2. Case 2

**Figure 2:**
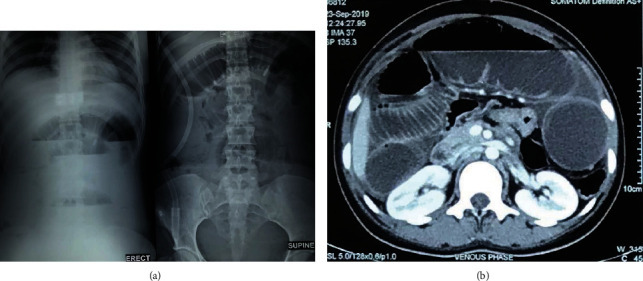
(a) X-ray showing air fluid levels in the left and dilated loops of small bowel with prominent valvulae conniventes suggestive of small bowel obstruction. (b) CECT scan showing dilated bowel loops suggestive of intestinal obstruction.

A 22-year-old female was admitted with a history absence of passage of stool and flatus accompanied by abdominal distension for 3 days and 1 episode of vomiting. Laboratory reports were normal except for a low hemoglobin level (9.9 gm/dL) and increased white blood cells (14,700) and platelet (745,000) counts. ABG showed evidence of acidosis with a pH of 3.99. Ultrasonography (USG) abdomen and pelvis and X-ray abdomen (Figure 2(a)) showed features of small bowel obstruction, while ECG showed sinus tachycardia (141/min). CECT of the abdomen (Figure 2(b)) revealed superior mesenteric artery occlusion. An echocardiographic evaluation was unremarkable. With the diagnosis of complete bowel obstruction with acute mesenteric ischemia due to SMA thrombosis, the patient was immediately transferred for surgery, and exploratory laparotomy with resection of gangrenous bowel and jejuno-ileal anastomosis was performed. Intraoperative finding was dilated gangrenous jejunal and ileal loops 50 cm distal from DJ flexure to 70 cm proximal to ileo-caecal (IC) junction. The patient improved postoperatively and was discharged on warfarin.

### 3.3. Case 3

A 47-year-old female presented to the emergency department with the history of acute-onset abdominal pain initially in the epigastric region which later generalized, along with abdominal distension. On examination, the abdomen was tense, distended with tenderness in the epigastric region, rebound tenderness, and reduced bowel sounds. Laboratory investigations were normal except elevated white cell counts of 18,700/mm^3^. An ABG analysis revealed a pH of 7.483 with lactate levels of 2.3 mmol/L, while an ECG showed sinus tachycardia (116/min). Echocardiography evaluation showed no clots or vegetations. CECT of the abdomen revealed findings suggestive of thrombosis in common hepatic artery and jejunal and ileal branches of superior mesenteric artery with an infarct in lower pole of right kidney. The patient was initially treated conservatively with anticoagulants due to refusal of surgery. With increasing abdominal pain, the patient was counseled and she underwent exploratory laparotomy with resection of gangrenous bowel and jejuno-ileal anastomosis. Intraoperative findings were gangrenous 110 cm of the small intestine extending from 70 cm distal to DJ flexure and a near perforation in the distal third of the resected segment. Postoperative, the patient was managed conservatively with anticoagulants for the renal infarct. Her condition improved and she was discharged home.

### 3.4. Case 4

A 39-year-old man was admitted to the hospital with a history of abdominal pain and distension accompanied by vomiting and an absence of passage of stool. Examination revealed distended abdomen with tenderness in all quadrants, guarding, and absence of bowel sound. Laboratory investigations reported a hemoglobin level of 9 gm/dL, white cell count of 13,000/mm^3^, and a platelet level of 706,000/mm^3^. USG Doppler of the leg revealed the features suggestive of deep vein thrombosis (DVT) of left lower limb. ECG showed sinus rhythm. With the diagnosis of acute mesenteric ischemia with peritonitis due to gangrenous bowel with sepsis, patient was subjected to the surgery on the same day and exploratory laparotomy with resection of gangrenous small intestine, and jejunostomy and ileostomy were performed. Intraoperative finding was a gangrenous small bowel extending 80 cm from DJ flexure to 20 cm proximal to IC junction. With continuous monitoring during the postoperative period and improvement in his condition, a follow-up jejuno-ileal anastomosis was performed. Later, as the patient's condition worsened, reexploration and double barrel jejunostomy and ileostomy were performed with intraoperative finding of leakage over the anastomotic site in the anterior and posterior aspect. With further improvement on the postoperative period, the patient was finally discharged on tab warfarin and was kept on follow-up.

### 3.5. Case 5

A 64-year-old man with a previous history of SMA thrombosis under warfarin, self-discontinued 5 days back, presented with a history of abdominal pain, vomiting, and no passage of stool or flatus. On abdominal examination, generalized tenderness, guarding, and an absence of bowel sound were noted. ECG revealed findings suggestive of atrial fibrillation. USG scan of the abdomen was normal; however, laboratory results showed a white cell count of 2,600/mm^3^ and a prothrombin time (PT)/international normalized ratio (INR) of 23/2.09. ABG report showed pH of 7.46 and lactate level of 2 mmol/L. With the diagnosis of chronic mesenteric ischemia, the patient underwent emergency laparotomy with jejunal resection with stapled functional end-to-end anastomosis with feeding jejunostomy. Intraoperative findings revealed stricture and impending jejunal perforation 70 cm distal to DJ flexure. However, the patient's condition did not improve postoperatively, and he succumbed to death 1 week after the operation due to refractory shock.

### 3.6. Case 6

A 45-year-old male was admitted to the hospital with chief complaints of periumbilical pain and passage of black-colored loose stool. On examination, his vitals were stable but the radial pulse on left forearm was feeble compared to his right forearm. Laboratory investigations were normal except for the white blood cell count of 18,600/mm^3^. Fecal stool occult blood test (FOBT) was positive. CT angiogram of left upper limb showed narrowing of vessel caliber, from the distal part of left axillary artery to proximal two-third of brachial artery with faint opacification of distal radial artery. Similarly, CT angiogram of the abdomen and pelvis including the thorax revealed eccentric thrombus in the arch and proximal descending part of thoracic aorta along with superior mesenteric artery thrombus, distal to the origin of proximal jejunal branches with edematous and reduced enhancing of ileal loops. Hence, with the diagnosis of acute mesenteric ischemia due to SMA thrombosis, the patient was immediately rushed to the operation theater, and emergency laparotomy was performed with resection of gangrenous jejunum and ileum with a double barrel jejuno-ileostomy. Intraoperative findings revealed gangrenous large segment of small bowel from 30 cm distal to DJ flexure to 20 cm proximal to IC junction. The patient improved on the postoperative period, was discharged, and readmitted later for ileostomy reversal.

### 3.7. Case 7

A 54-year-old man presented with chief complaints of acute-onset abdominal pain, vomiting, and not passing stool. Laboratory investigations were normal, ECG showed sinus rhythm, and ABG findings were not significant (pH: 7.45; lactate: 1.4). However, CECT of the abdomen and pelvis showed intraluminal filling defect in celiac trunk which was suggestive of thrombosis. With no further delay, the patient was admitted and rushed for operation on the same day, and exploratory laparotomy with resection and anastomosis was performed. Intraoperative findings showed a gangrenous patch of the small intestine, approximately 100 cm, extending distal from 80 cm of DJ junction. Later, the patient improved postoperatively and was discharged with regular follow-ups.

### 3.8. Case 8

An 86-year-old female was admitted with a history of pain in the abdomen. On examination, atrial fibrillation was found. Lab investigations were normal except white cell counts of 22,000/mm^3^. ECG showed atrial fibrillation and an ABG revealed normal findings except for an increase in lactate level of 2.5. Echocardiography showed dilated left atrium (LA) and moderate mitral regurgitation (MR), and a USG examination showed features suggesting chronic DVT and peripheral arterial disease. CECT of the abdomen and pelvis showed total occlusion of distal half of superior mesenteric artery and the right common iliac artery extending to the internal and external iliac artery. The patient was counseled of the need for surgery, its outcomes, and prognosis. However, she refused it, and hence, she was managed conservatively with anticoagulants and was discharged on patient request. The patient was subsequently lost for follow-up.

### 3.9. Case 9

A 70-year-old man was admitted with a history of abdominal pain which was central and radiating to the back. On abdominal examination, there was tenderness on the left iliac fossa and bowel sounds were normal. Laboratory investigations were normal except for a hemoglobin of 10.4 gm/dL. ECG showed normal sinus rhythm and an ABG revealed a pH of 7.46 and a lactate level of 0.4 (normal level). CECT of the abdomen revealed the features suggestive of omental infarction with suspicious filling defect in one of the left branches of SMA. Mesenteric angiography then showed approximately 50% stenosis of SMA region and complete occlusion of distal portion of first left branch of SMA (jejunal artery). With the diagnosis of Chronic mesenteric ischemia, the patient was managed conservatively first with injection heparin which was later switched to oral warfarin. With improvement in his condition, the patient was discharged on warfarin with further follow-ups.

### 3.10. Case 10

A 23-year-old lady was admitted in the hospital with the diagnosis of postsplenectomy pancreatic fistula with acute portal vein thrombosis. She was a known case of idiopathic thrombocytopenic purpura. CECT of the abdomen and pelvis revealed thrombosis of portal vein and superior mesenteric vein, with long segment diffuse edematous wall thickening of the distal ileum sparing the terminal ileum likely due to small bowel ischemia. The patient was managed conservatively and was discharged on warfarin.

## 4. Discussion

Mesenteric ischemia has a prevalence of about 1 in 1000 hospital admissions [8]. Its peak incidence is at the 6th to 7th decade of life with an equal prevalence among both males and females [1].  Table 1 shows the mean age and prevalence of mesenteric ischemia among male and female in our study. Among the various risk factors of mesenteric ischemia, age > 60 years is considered to be a significant risk factor [9]. However, more than 50% of our cases (6 out of 10) occurred in patients of age less than 60 yrs. In contrast to this, advanced age does have an effect on the mortality rate as exemplified in our case series, which is in agreement with studies that have shown survivors of mesenteric ischemia to be younger [10, 11].

The presenting signs and symptoms of acute mesenteric ischemia are vague, with the classical complaint being abdominal pain out of proportion to physical findings and frequently accompanied by vomiting. In addition to this, it can present with signs and symptoms of bowel obstruction as well [2]. Once bowel infarction has occurred, peritonitis and shock become the dominant clinical feature [12, 13]. One might rely on the absence of bowel sounds to make a diagnosis, but bowel sounds tend to be absent only when infarction sets in [1]. In our case series, 5 of 9 patients with arterial occlusion presented with initial features similar to bowel obstruction (vomiting, unable to pass stool, and abdominal distension), and 1 of 9 patients had passage of black-colored stool.

The presence of cardiac findings supports the diagnosis of mesenteric ischemia in patients presenting with features described above. It has also been correlated with increased mortality with reviews showing up to 70% lethality in cases of mesenteric ischemia with atrial fibrillation [14]. In our study, 3 out of 9 patients with mesenteric ischemia had atrial fibrillation (AF) with mortality in 2 of them (66.66% mortality rate).

Leukocytosis, increased serum lactate, and metabolic acidosis with increased anion gap have been used as laboratory adjuncts for the diagnosis of intestinal ischemia [2, 15]. Though these findings are usual in an ideal case, normal pH and metabolic alkalosis are also possible findings [12]. In our case series, we found neutrophilic leukocytosis to be the most consistent laboratory abnormality, being present in 7 of 10 cases. One of the patients had leukopenia rather than leukocytosis, while 2 had normal WBC counts. This illustrates the role of preoperative leucopenia as a mortality predictor in cases of acute mesenteric ischemia who undergo surgical management [16]. In addition to this, we found metabolic acidosis as an inconsistent marker with the majority of them having a normal pH. Lactate levels were increased (with an average level of 2 mmol/L) in five out of six patients in whom it was measured. Hemoconcentration was not present in any of the cases, though it is possible because of third spacing of fluid [2]. The laboratory findings and mortality outcomes of patients in this study have been summarized in Table 2.

Apart from the laboratory markers, various radiological techniques are used in the diagnosis of mesenteric ischemia. Pneumatosis intestinalis and thumb-print sign (focal edematous haustra) are some of the signs suggestive of bowel infarction on a plain abdominal X-ray. Nonspecific findings such as adynamic and mechanical small bowel obstruction can also be seen on plain films of mesenteric ischemia [13], which has been demonstrated in our case series. Similarly, some findings in noncontrast computed tomography (NCCT) scan, which has a sensitivity of 39% that supports a diagnosis of mesenteric ischemia, are pneumatosis intestinalis, presence of mesenteric or portal venous gas, and focal bowel wall thickening. Thus, plain abdominal X-ray and NCCT are rarely diagnostic [13] and are more useful in ruling out alternative causes of acute abdomen. Modern settings, therefore, utilize dynamic contrast-enhanced computed tomography (CECT), which has improved diagnostic sensitivity, as the go to test in diagnosing mesenteric ischemia [17, 18]. While most cases are picked up by the CECT scan, mesenteric angiography is used in cases of equivocal findings [3, 19, 20]. In our case series, when the CECT scan revealed only suspicious findings, mesenteric angiography was resorted to, in order to confirm the diagnosis as well as to locate the level and extent of obstruction.

Splanchnic circulation receives approximately 25% of resting cardiac output, the major portion of which is distributed to the mucosa and submucosa (70% of mesenteric blood flow) [2, 5]. This causes the mucosa to be affected early by ischemia. The small intestine can withstand up to 6 hours of ischemia before undergoing necrosis [21]. Because of this, FOBT is usually negative in the early stages until mucosal infarction has occurred [22]. Hence, in a suspected case, the absence of a positive FOBT does not rule out mesenteric ischemia but its presence makes the diagnosis more likely as demonstrated in our case series.

Mesenteric ischemia can be either acute or chronic. Acute mesenteric ischemia can further be classified as being due to arterial embolism (40-50%), arterial thrombosis (25%), nonocclusive mesenteric ischemia (20%), and venous thrombosis (5-10%) [2, 3]. Emboli, whose source is mainly cardiac, gets lodged most frequently in the middle colic artery, a branch of SMA, while thrombosis occurs mostly at the origin of superior mesenteric artery (SMA). Hence, the extent of infarction is more in arterial thrombosis [2]. This also signifies that SMA is the most common artery to get involved in mesenteric ischemia. The same has been reported in our case series with 8 of 10 cases affecting SMA. Causes of mesenteric vein thrombosis include hypercoagulable states whether inherited or acquired and intra-abdominal pathological conditions like malignancy, pancreatitis, and intra-abdominal sepsis [2]. In our case series, the presence of a pancreatic fistula as an intra-abdominal pathology probably led to thrombus formation.

Specific etiology demands specific management. Mesenteric arterial occlusion either by a thrombus or an embolus is treated with surgical laparotomy and/or thrombectomy/embolectomy or thrombolysis, the choice of which depends on the time course, extent, comorbidities, presence of symptoms of acute abdomen, and hemodynamic stability of the patient. All that mesenteric vein thrombosis requires is anticoagulation unless symptoms keep persisting which needs exploratory laparotomy. Two of our cases with SMA thrombosis without signs of acute abdomen with maintained hemodynamic stability were managed conservatively. Once intestinal ischemia is suspected, IV fluid resuscitation (because of third spacing), IV broad-spectrum antibiotics (because of bacterial translocation from the gut—“the gut-lymph theory”), and anticoagulation with heparin should be started immediately [2, 6, 20, 23]. The presence of peritoneal signs mandates early laparotomy, in order to avoid dreadful complications like septic shock and hypothermia [9]. Out of 10 cases, 7 patients underwent laparotomy, while 3 patients were treated conservatively. All of the 7 patients who underwent laparotomy had some segment of gangrenous bowel removed. Most of them underwent surgery within 24 hrs of presentation except 1 case. Because of the presence of signs of peritonitis with unstable vitals, 2 of our cases underwent exploratory laparotomy without undergoing a prior CECT scan. Regardless of etiology, the prognosis depends upon the presence and extent of bowel infarction [24]. Higher American Society of Anesthesiology (ASA) grade, older age (>70 years), and higher lactate levels have also been found to correlate with a higher mortality rate [11]. Similarly, postoperative anastomotic leakage can lead to a higher rate of reoperation and a higher mortality rate [25]. Studies have shown a diverting stoma to be a safer option with a shorter operation time and an early resumption of oral intake [26]. Our case series had a mortality rate of 20% with older age (>60 yrs), the presence of atrial fibrillation, leukopenia, and complications after surgery, including anastomotic leakage as a major one, being associated with a higher mortality.

## 5. Conclusion

A clinical diagnosis of acute mesenteric ischemia should be considered in patients presenting with features suggestive of bowel obstruction along with AF. While a positive fecal occult blood test further points towards the diagnosis, it is likely to be positive only after mucosal infarction sets in. In those with a diagnostic dilemma, we recommend using contrast enhanced computed tomography to aid in the diagnosis; however, in those presenting with features of peritonitis and unstable vitals, exploratory laparotomy without a prior CT scan is justified. Since time is everything in this disease, early exploratory laparotomy (within 24 hours) can be a life-saving procedure as exemplified in our case series. Postoperative complications especially anastomotic leakage are associated with a higher mortality rate despite early diagnosis and treatment. Therefore, the decision of a primary anastomosis vs. a diverting stoma should be made tactfully.

## Figures and Tables

**Table 1:** Data averages of patients.

Mean age	55 yrs
M:F	3 : 2
Mortality rate at discharge	20%
Elevated lactate levels	5/6 (83%)
Atrial fibrillation	3/9 (33%)

M: male; F: female; P: presence of atrial fibrillation; N: absence of atrial fibrillation; N/A: not available; D: dead; A: alive at discharge.

**Table 2:** Summary of patients' demography, laboratory findings, treatment, and outcomes.

Case	Age	Sex	ECG	WBC (counts/mm^3^)	pH	Lactate (mmol/L)	Clinical features	Operative findings	Management	Mortality
1	76	M	P	16000	7.418	2	Pain abdomen; not passing stool; vomiting 2 days back; absent bowel sounds	Gangrenous bowel 70 cm distal from DJ flexure to hepatic flexure	Operative	D
2	22	F	N	14700	7.399	N/A	Unable to pass stool and flatus for 3 days; vomiting 1 episode	Dilated gangrenous jejunal and ileal loops 50 cm distal from DJ flexure to 70 cm proximal to IC junction	Operative	A
3	47	F	N	18700	7.483	2.3	Abdominal pain-epigastric region later generalized, associated with abdominal distention	70 cm distal from DJ flexure, 110 cm segment of the small intestine was ischemic	Operative	A
4	39	M	N	13000	N/A	N/A	Abdominal pain; abdomen distension; not passed stool; vomiting	Gangrenous small bowel extending distal 80 cm from DJ flexure to 20 cm proximal from IC junction	Operative	A
5	64	M	P	2600	7.46	2	Abdominal pain and vomiting; not passing stool/flatus	Chronic mesenteric ischemia with stricture and impending jejunal perforation 70 cm distal to DJ flexure	Operative	D
6	45	M	N	18600	N/A	N/A	Periumbilical pain and passage of black loose stool	Large segment of the small bowel involving from 30 cm distal to DJ flexure to 20 cm proximal to IC junction was gangrenous	Operative	A
7	54	M	N	9410	7.45	1.4	Abdominal pain and not passing stool with vomiting	Gangrenous patch of approx. 100 cm extending distally from 80 cm of DJ junction	Operative	A
8	86	F	P	22000	7.449	2.5	Abdominal pain	N/A	Patient denied operative treatment and was managed conservatively	A
9	70	M	N	8400	7.46	0.4	Central pain radiating to back; soft abdomen, nondistended	N/A	Conservative	A
10	23	F	N/A	15690	N/A	N/A	A case of postoperative pancreatic fistula with abdominal pain and soft abdomen passing stool	N/A	Conservative	A

## Data Availability

Due to privacy purpose, any data not mentioned in the article will remain confidential.
